# Mechanisms of bystander effects in retinal pigment epithelium cell networks

**DOI:** 10.1038/cddis.2017.449

**Published:** 2017-10-05

**Authors:** Masaaki Ishii, Bärbel Rohrer

**Affiliations:** 1 Department of Ophthalmology, Medical University of South Carolina, Charleston, SC 29425, USA; 2 Department of Neuroscience, Medical University of South Carolina, Charleston, SC 29425, USA; 3 Ralph H Johnson VA Medical Center, Charleston, SC 29401, USA

The bystander effect is part of a cell’s cell–cell communication repertoire, and refers to the observation that biological effects can be observed in cells that are not directly targeted. This type of communication can be mediated by intercellular communication via gap junctions (GJs), or by messengers released from targeted cells that travel across the plasma membrane or are secreted extracellularly.^1^ This phenomenon has been studied in radiobiology and was first reported in 1992.^2^ However, it has since been observed to have a role in various types of cells and tissues. Overall, these results have led Brooks^3^ to conclude that the ‘bystander effects suggest that organs respond as a unit and are not just a bag of individual cells acting independently’.

Ishii and Rohrer^4^ have studied bystander effects in networks of retinal pigment epithelium (RPE) cells, using high-resolution imaging. The RPE lends itself to this kind of two-dimensional analysis, since it is composed of a single layer of hexagonal cells that are connected by tight and adherence junctions to establish barrier function, and GJs to mediate intercellular communication,^5^ features that can be replicated *in vitro*. The RPE is of interest in diseases such as age-related macular degeneration (AMD), macular edema and diabetic retinopathy, whose disease processes involve oxidative stress. The authors argued that since the RPE is a highly coupled network, any individual cell will be significantly affected by the behavior of its neighbors; and second, they suggested that the susceptibility of a given cell to bystander signals is dependent upon its prior metabolic history and mitochondrial health.

Cell–cell communication was examined using live-cell imaging in response to blue-laser spot simulation of individual cells. Blue light was chosen since it is assumed as a risk factor for AMD,^6^ it induces reactive oxygen species (ROS) production and lipid peroxidation in RPE cells^7^ resulting in apoptotic cell death;^8^ and it can cause mitochondrial damage.^9^ As cellular readouts of cell–cell communication and mitochondrial health, the characteristics of induction and transfer of ROS and calcium ions (Ca^2+^) to connected neighboring cells and their mitochondrial membrane potential (*ψ*_m_) was examined (Figure 1); readouts of long-term consequences included the analysis of cell death.

Stimulation of a single, randomly selected RPE cell within an established network with blue light (488 nm laser; 20 ms flashes at 1 Hz, 38 kw/cm^2^ intensity) resulted in the rapid generation of a continuous ROS signal (hydrogen peroxide (H_2_O_2_), H_2_DCFDA) followed by the generation of hydroxyl radicals (CellRox-Green) in the stimulated cell. This was followed by a transient increase first in cytosolic and subsequently mitochondrial Ca^2+^ (Fluo8). Upon transfer of Ca^2+^ into the mitochondria, mitochondrial membrane potential changes (*ψ*_m_; tetramethylrhodamine-methyl ester (TMRM)) were triggered, starting with a transient increase in membrane hyperpolarization followed by membrane depolarization. On the basis of the known biology of oxidative stress, these changes were predictable. However, what makes this manuscript exciting and novel, is the analysis of the bystander effect in connected cells.

Each RPE cell is coupled to six neighboring cells; however, signal transfer differed, depending on the metabolite analyzed (Figure 1). ROS signals were found to spread rapidly and radially, leading to long-lasting changes in ROS in all connected cells. In contrast, the Ca^2+^ signal was transmitted to only certain neighboring cells, and changes in *ψ*_m_ was restricted to cells that received the Ca^2+^ signal.

To determine the potential mechanism of signal transfer, GJ blockers 18*β*-glycyrrhetinic acid (*β*GA) or 1-octanol were added. While the transfer of the ROS signal was not inhibited by GJ blockers, the transfer of the Ca^2+^ signal and the subsequent changes in *ψ*_m_ were completely eliminated.

Calcium is an essential intracellular signaling molecule, and its levels are tightly regulated. Relevant for this study, mitochondria and the endoplasmic reticulum (ER) have important roles in Ca^2+^uptake and release, and a link between Ca^2+^ dysregulation, mitochondrial alterations and cell death has been evident in many disorders. Also, melanin, a hallmark of RPE, binds Ca^2+^. In the current study, Ca^2+^ levels were found not to be uniform in the resting RPE network. Interestingly, both the ROS increase and the *ψ*_m_ amplitude elicited by the bystander effect in a given surrounding cell exhibited a linear correlation with the cell’s baseline Ca^2+^ concentration. Likewise, Ca^2+^ levels were found to be negatively correlated with the amount of pigmentation present in a cell, supporting a role of melanin in the regulation of Ca^2+^ homeostasis.^10^ Since Ca^2+^ homeostasis is essential for cell survival, the RPE networks were analyzed over a 20 h period to examine the long-term consequences of photo-oxidative stress. Laser stimulation of a single RPE cells was found to induce cell death (To-Pro3) in approximately half the cells present in the recording window. Application of *β*GA reduced cell death to ~10%. Intriguingly, and similar to the short-term results on mitochondrial health, the higher the Ca^2+^concentration at baseline, the greater the risk for the cell to undergo future cell death.

The ER and mitochondria as Ca^2+^-storing organelles, and mitochondria as the major checkpoint for apoptosis have been studied extensively. Ca^2+^ transfer between the cytoplasm and the ER is mediated via the sarco/ER Ca^2+^-ATPase (SERCA), its ER release is mediated via the activation of either IP3 or ryanodine receptors, and mitochondrial uptake is accomplished via a Ca^2+^-uniporter.^11^ In the RPE network, a critical role for the ER could be identified, as the SERCA inhibitor thapsigargin (Sigma Aldrich) reduced cell death to <10%. ER Ca^2+^ release was mediated by ryanodine receptors, as the IP3-receptor antagonist – 2-aminoethoxydiphenyl borate (2APB) did not inhibit cell death, whereas the ryanodine-receptor antagonist, dantrolene did. Finally, an essential role for mitochondrial Ca^2+^ release was verified by demonstrating that treatment with the mitochondrial Ca^2+^ uptake inhibitor Ru360 completely inhibited cell death. Together, these results suggest that Ca^2+^-mediated cell death in RPE networks is mediated by ER-mitochondria Ca^2+^ transfer.^11^

The results presented by Ishii and Rohrer^4^ convincingly demonstrate that local photo-oxidative stress in a donor RPE cell can trigger cellular damage that require a dual-hit of ROS and Ca^2+^-related signals. The identified metabolic signature of elevated baseline Ca^2+^ levels should be further investigated as a contributing factor to disease processes in networks of cells in which initial damage seems to occur in susceptible, and is delayed in more resilient areas.

## Figures and Tables

**Figure 1:**
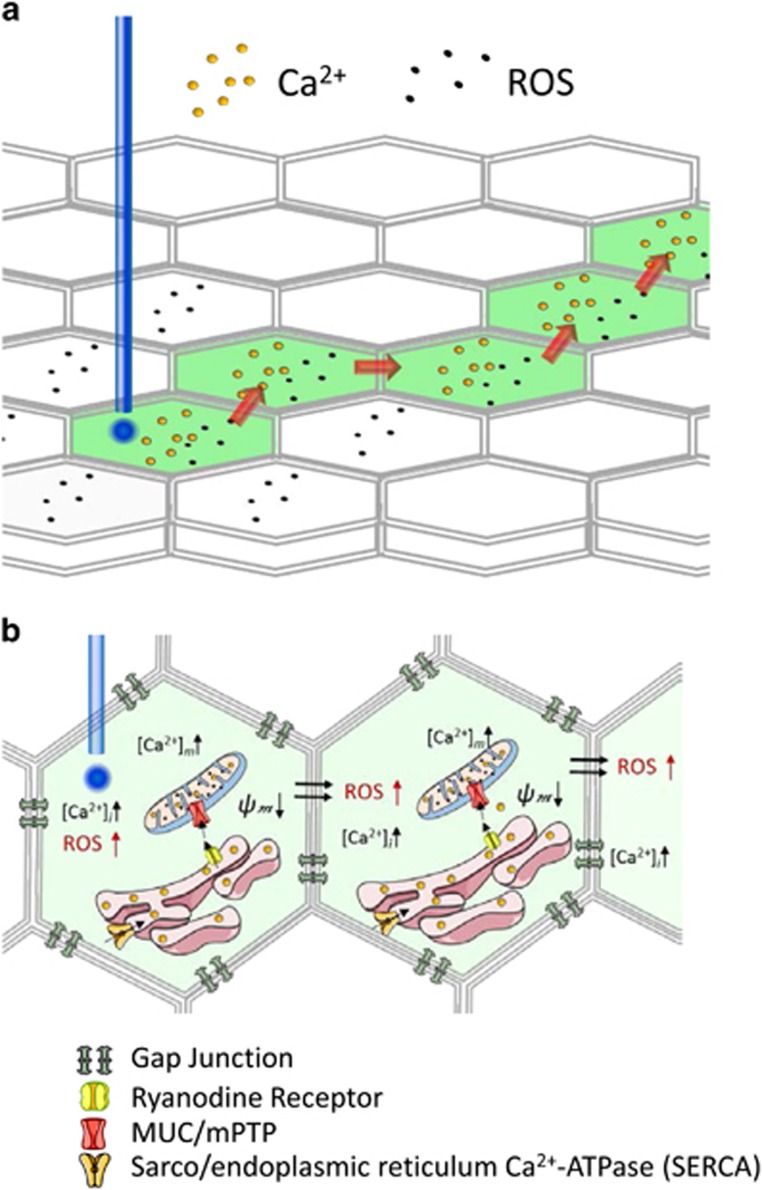
(**a**) Rapid information transfer mediated by the bystander effect differs for ROS and Ca^2+^. Information related to ROS (hydrogen peroxide, H_2_O_2_, and hydroxyl radicals,·OH−) peaks and spreads rapidly in a radial manner from the stimulated cell (blue-light stimulus) to its neighbors, leading to long-lasting changes in ROS in all connected cells. In contrast, the transfer of the calcium (Ca^2+^) signal was not uniform, but rather was restricted to only certain neighboring cells, with concomitant changes in *ψ*_m_ only being elicited in cells that also received the Ca^2+^ signal. (**b**) Cell death as a long-term consequence of photo-oxidative stress-mediated bystander effect in RPE network. In the central cell, photo-stimulation of the mitochondrial network was found to lead to an increase in ROS and mitochondrial Ca^2+^ as well as a loss in mitochondrial membrane potential (*ψ*_m_), leading to rapid cell death. Local oxidative stress in a donor cell subsequently triggered metabolic changes in certain connected recipient cells, an effect that required gap junction (GJ) communication and an ROS-Ca^2+^ dual-hit, resulting in slow cell death. The transfer of the Ca^2+^ signal to neighboring cells requires GJ communication; the transfer of the ROS signal does not. Cell death triggered by mitochondrial Ca^2+^ overload was mediated by endoplasmic reticulum (ER)-mitochondria Ca^2+^ transfer, involving Ca^2+^ uptake via the SERCA/ER ATPase, ER efflux via the ryanodine receptor (RyR), and Ca^2+^ uptake into the mitochondria via the uniporter or the mitochondrial permeability transition pore (MCU, mPTP)

## References

[bib1] Hall EJ Health phys 2003; 85: 31–35. 12852468 10.1097/00004032-200307000-00008

[bib2] Nagasawa H, Little JB Cancer Res 1992; 52: 6394–6396. 1423287

[bib3] Brooks AL Hum Exp Toxicol 2004; 23: 67–70. 15070062 10.1191/0960327104ht419oa

[bib4] Ishii M, Rohrer B Cell Death Discov 2017; 3: 16071. 28179989 10.1038/cddiscovery.2016.71 PMC5292780

[bib5] Quan V et al Mol Vis 2010; 16: 1343–1352. 20664797 PMC2905638

[bib6] Kijlstra A et al Prog Retin Eye Res 2012; 31: 303–315. 22465791 10.1016/j.preteyeres.2012.03.002

[bib7] Nakanishi-Ueda T et al Free Radic Res 2013; 47: 774–780. 23898883 10.3109/10715762.2013.829570

[bib8] King A et al Photochem Photobiol 2004; 79: 470–475. 15191057 10.1562/le-03-17.1

[bib9] Godley BF et al J Biol Chem 2005; 280: 21061–21066. 15797866 10.1074/jbc.M502194200

[bib10] Bush WD, Simon JD Pigment Cell Res 2007; 20: 134–139. 17371440 10.1111/j.1600-0749.2007.00362.x

[bib11] Rizzuto R et al Biochim Biophys Acta 2009; 1787: 1342–1351. 19341702 10.1016/j.bbabio.2009.03.015 PMC2730423

