# Correction: Mechanisms of bystander effects in retinal pigment epithelium cell networks

**DOI:** 10.1038/s41419-018-0831-3

**Published:** 2018-07-16

**Authors:** Masaaki Ishii, Bärbel Rohrer

**Affiliations:** 1 Department of Ophthalmology, Medical University of South Carolina, Charleston, SC 29425 USA; 2 Department of Neuroscience, Medical University of South Carolina, Charleston, SC 29425 USA; 3 Ralph H Johnson VA Medical Center, Charleston, SC 29401 USA

Correction to: *Cell Death & Disease* ; 10.1038/cddis.2017.449; published online 5 Oct 2017.

The PDF and HTML versions of the article have been updated to include the Creative Commons Attribution 4.0 International License information.

